# Aortic Graft Infection as a Cause of Multiple Brain Infarcts

**DOI:** 10.1155/2012/575169

**Published:** 2012-08-30

**Authors:** Vassiliki Tsirka, Jelena Maletic, Panagiotis Ioannidis, Dimitrios Karacostas

**Affiliations:** B' Neurological Department, AHEPA University Hospital, 54636 Thessaloniki, Greece

## Abstract

Brain embolism of cardiac origin is common in clinical practice. However, embolic brain infarcts due to aortic graft infection are very rare. We present a case of a 53-year-old woman with multiple brain infarcts, following an infection of ascending aortic graft. She was presented with fever and acute onset neurological deficit, and she had a previous history of replacement of ascending aorta with a prosthetic graft, because of aortic aneurysm 2 years before her admission. The patient had positive blood cultures and echocardiographic evidence of vegetation in the graft aortic joint, nearby the aortic valves. Despite the severe clinical condition and the poor prognosis, because of the coexistence of cardioembolism and aortic graft infection, our patient had a good outcome with conservative treatment and she will be considered for surgical graft replacement after her full recovery.

## 1. Introduction

Cardioembolic stroke represents the one fifth of ischemic strokes' causes. Among the most common embolic risk disorders are reported the atrial fibrillation, prosthetic heart valve, rheumatic mitral stenosis, infective endocarditis, intracardial tumors, dilated cardiomyopathy, and recent myocardial infarction [1]. However, there are scattered reports of brain embolism due to aortic graft infections [2] as a late onset complication. 

The incidence of graft infections following surgery of the thoracic aorta is 0.9% to 1.9% [3] and correlates with a high mortality rate ranging from 25% to 75% [4]. Although most graft infections occur in the first month postoperative, they may emerge some years after prosthetic graft implantation [5] due to hematogenous seeding as well as bacteria harbored in atherosclerotic plaques [6].

The most frequent responsible infectious organisms are *Staphylococcus aureus, Staphylococcus epidermidis,* and rarely *Pseudomonas* and fungus [5]. The clinical manifestation usually takes the form of a systemic inflammatory response syndrome with fever, leukocytosis, and tachycardia, although sometimes the clinical presentation can be insidious. 

Cardiogenic emboli are distributed evenly through the body circulation, potentially affecting many organs, with a high prevalence, around 80% [7], involving the brain, resulting in the clinical manifestation of stroke. The main characteristic of brain cardioembolism is the presence of multiple infarcts involving different cerebral arterial territories. Because of the specific pathophysiological mechanism of ischemia, these infarcts are highly associated with hemorrhagic transformation and with a clinical course of rapid resolution of the neurological deficit. 

## 2. Case Report 

A 53-year-old woman was admitted to our department because of acute neurological deficit. She had a past medical history of replacement of an ascending aorta with a prosthetic graft due to aortic aneurysm 2 years before her present admission along with a known, bicuspid aortic valve without functional repercussion.

Four days before her admission, she complained about abdominal pain, nausea, gastrointestinal disturbances, and high fever up to 40°C, followed by acute aphasia and right hemiplegia.

On admission her vital signs included blood pressure 130/80 mmHg, heart rate 90 bpm, temperature 38.5°C, oxygen saturation of 98%, and respiratory rate of 14 breaths per minute. Her initial electrocardiogram (ECG) had no signs of acute abnormalities.

The neurological status included a Glasgow Coma Scale score of 11 (3-1-6), severe motor aphasia, right hemiplegia with hemineglect, right homonymous hemianopsia, left-side gaze deviation, right central facial palsy, and ipsilateral Babinski sign.

The patient's complete blood count revealed elevated white blood cell count to 17960/mm^3^ with C-reactive protein level of 16 mg/dL and sedimentation rate of 84 mm. There was a moderate elevation of liver function tests (alanine transaminase) ALT (52 U/L, aspartate transaminase) AST (42 U/L). All other biochemistry tests were within normal range.

**Figure 1:**
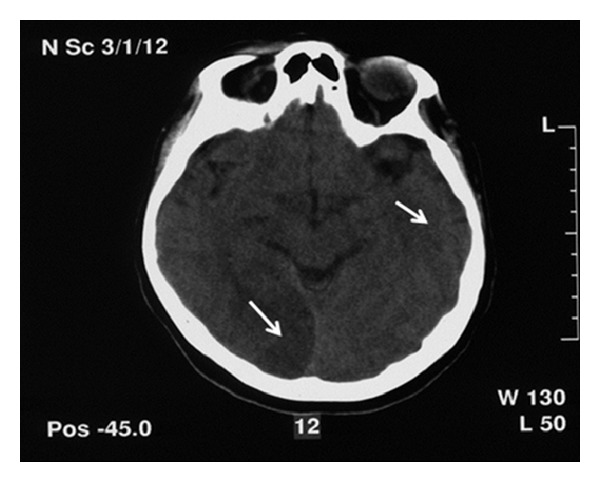
First brain CT scan with multiple infarcts.

**Figure 2:**
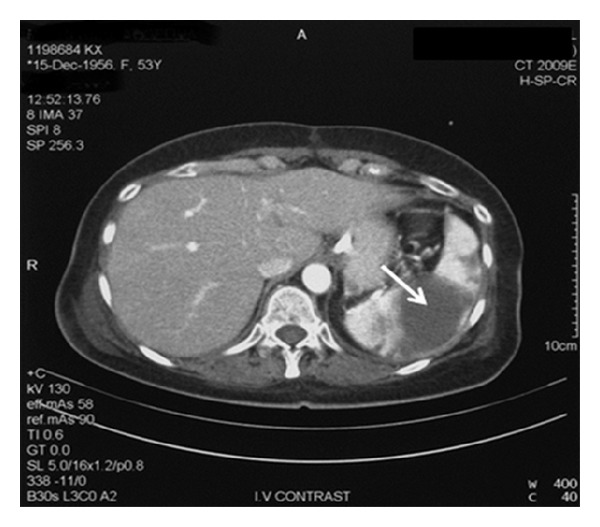
Abdominal CT with splenic infarcts.

The initial brain computed tomography (CT) (Figure 1) revealed multiple infra- and supratentorial infarcts in both hemispheres more prominent on the left. Thoracic and abdominal CT displayed pleuritic infusion and multiple splenic infarcts (Figure 2). 

**Figure 3:**
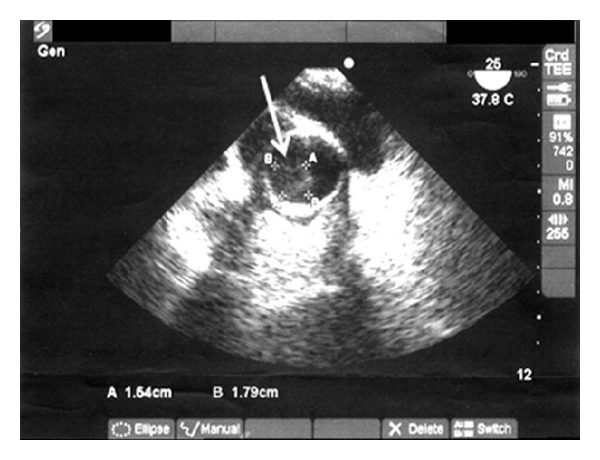
Transesophageal echocardiography with vegetation of aortic graft joint.

The transesophageal echocardiography showed a pedicle-like movable formation on the aorticgraft joint near the aortic valves, with dimensions 1.54 × 1.79 cm (Figure 3). Blood culture was positive for *Staphylococcus aureus,* and the patient was started on intravenous beta-lactam antibiotic with beta-lactamase inhibitor (piperacillin/tazobactam) according to the antibiogram in combination with gentamicin, and after some days she received vancomycin.

**Figure 4:**
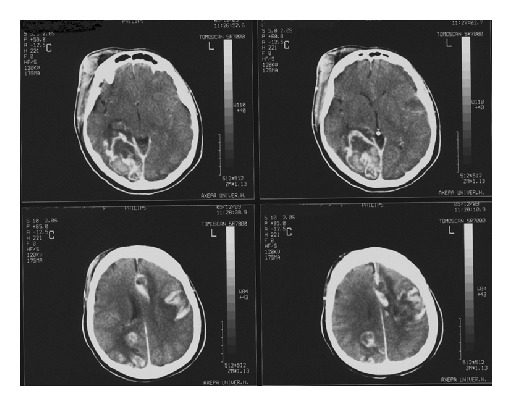
Brain CT scan with hemorrhagic transformation of infarcts.

**Figure 5:**
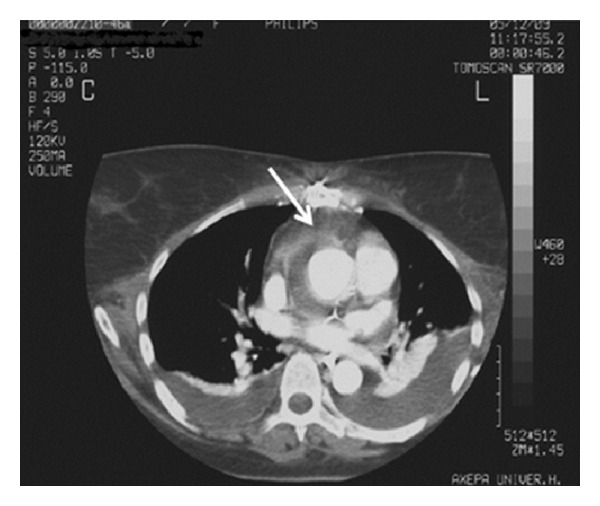
Thoracic CT with mediastinitis.

However, the patient had fluctuating fever and she exhibited a neurological deterioration after 1 week with decline of consciousness level and the brain CT demonstrated hemorrhagic transformation of the cerebral infarcts (Figure 4). The follow-up thoracic CT showed mediastinitis located around the graft region (Figure 5).

**Figure 6:**
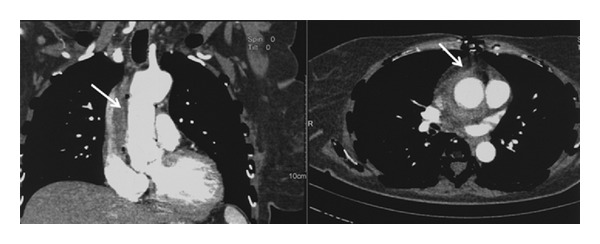
Thoracic CT scan with mediastinitis in regression.

 During the course of the illness, there was a clinical amelioration, continuously improving neurological status with brain imaging showing regression of focal lesions. Simultaneously, the successive thoracic CTs demonstrated gradual resolvement of mediastinitis (Figure 6), and her blood chemistry and inflammatory indices progressively returned to normal with negative repetitive blood cultures.

After 3 months of hospitalization and completion of 6-week intravenous antibiotic administration, the patient achieved nearly full recovery with residual mild right hemiparesis and motor aphasia. The last transesophageal echocardiography, thoracic CT, and blood culture were normal. 

Due to her critical condition, she was not treated surgically and she had a satisfactory clinical recovery. Low molecular weight heparin (LMWH) was administered in order to prevent possible deep vein thrombosis. She received doxycycline as prophylactic antibiotic medication 2 months after her hospitalization, and she has continuous speech therapy and rehabilitation. She was consulted to be evaluated for graft replacement by cardiothoracic surgeons. She was not reoperated, but she was under observation, with special attention in any febrile condition with not evident cause.

## 3. Discussion

The incidence of cardioembolic strokes after infected vegetations of aortic implants is unknown, mainly because the frequency of graft infection as a late-onset complication is very low. Moreover, this is a very critical condition with high mortality and possibly the patients cannot evolve the whole spectrum of potential secondary events. The most impressive fact about the reported patient is that the first manifestation of the underlying condition was the acute presentation of neurological deficit and the incriminated cause was revealed during the detailed investigation.

Because of the rarity of such cases, there is no specific treatment protocol. There are many reports of possible surgical treatments for aortic graft infections [5, 8, 9] that could not be applied in this case because of the severity of patient's clinical condition. Although the treatment of most cases of cardioembolic strokes comprises the use of anticoagulants [10], the obvious choice in this situation was to follow the treatment protocol for cerebrovascular events secondary to infective endocarditis [11]. We proceeded with early administration of correct antibiotic therapy, withholding anticoagulation since there is no evidence of warfarin effectiveness as a secondary prevention in systemic embolisation, while there is an estimated high risk up to 40% of subsequent hemorrhagic transformation [12]. Additionally, in this case, the presence of multiple splenic infarcts, the hemorrhagic conversion of brain infarcts, and the isolation of *Staphylococcus aureus* as the responsible infectious agent [13] rendered the use of anticoagulation impossible.

This is a rare case of neurological complication after ascending aortic graft infection. The expected outcome of the patient, estimating the high mortality risk of both graft infection [4] and cardioembolism [12], was initially poor. Despite all odds, the therapeutic choices and possibly the absence of previous concomitant diseases led to a successful recovery. The patient will be considered for surgical graft replacement after her full recovery in order to prevent future, subsequent episodes.
